# Bimodality in pan-cancer proteomics reveals new opportunities for biomarker discovery

**DOI:** 10.1186/s13059-026-04190-z

**Published:** 2026-07-09

**Authors:** Wen Jiang, Damian Bikiel, Jan Zaucha, Jixin Wang, Sanddhya Jayabalan, Yongjin Li, Matthew Sung, James Harper, Jimmy Zhao, Krishna Bulusu, Wenyan Zhong

**Affiliations:** 1 Oncology Data Science, Oncology R&D, AstraZeneca, Gaithersburg, MD USA; 2 Oncology Data Science, Oncology R&D, AstraZeneca, New York, NY USA; 3 Oncology Data Science, Oncology R&D, AstraZeneca, Cambridge, UK; 4 Oncology Data Science, Oncology R&D, AstraZeneca, Waltham, MA USA; 5 Oncology Targeted Discovery, Oncology R&D, AstraZeneca, Waltham, MA USA; 6 Translational Medicine, Oncology R&D, AstraZeneca, Cambridge, UK; 7 Late Development Oncology, Oncology R&D, AstraZeneca, New York, NY USA

## Abstract

**Background:**

Bimodal protein expression, defined as the distribution of protein expression with two modes, is linked to phenotypic variation across various biological systems. To advance the identification of cancer biomarkers and targets for precision oncology beyond RNA-based studies, we developed a proteomics-specific bimodality model.

**Results:**

By analyzing proteomics data from various cancer types, 2401 tumor-associated bimodal proteins significantly linked to critical cancer pathways were identified, including amino acid metabolism, extracellular matrix-receptor interaction, and central carbon metabolism. Utilizing an AI-enhanced knowledge graph, we further delineated common patterns among pan-cancer tumor-associated bimodal proteins. A case study on *TROP2* in colon adenocarcinoma highlights up-regulation of *MYC* and *WNT*/β-catenin pathways and down-regulation of inflammatory pathways in *TROP2*-high groups.

**Conclusion:**

This research highlights the biological differences impacting cancer heterogeneity and vulnerability, ultimately aiding treatment decisions. Our findings illustrate the value of proteomics in uncovering novel biomarkers and advancing precision medicine, paving the way for multi-omics integration and clinical validation.

**Supplementary Information:**

The online version contains supplementary material available at https://doi.org/10.1186/s13059-026-04190-z.

## Background

Identifying cancer-associated targets and biomarkers from high-throughput genomics, transcriptomics, and proteomics is a fundamental process in precision oncology [1–6]. Traditionally, cancer biomarkers and oncogenes are discovered by comparing tumor samples with matched healthy tissues [7–10]. More recently, the identification of distinct gene programs activated in heterogeneous tumor tissues—manifested as bimodal expression peaks in RNA sequencing (RNA-seq) profiles—has emerged as an alternative approach [11–13]. The utility of bimodal genes as biomarkers lies in their ability to classify patients into discrete subgroups; for example, receptor *ESR1*’s bimodal expression stratifies breast cancer patients into ER-positive and ER-negative subgroups, fundamentally guiding the therapeutic strategies [14, 15].

Numerous studies have applied bimodality detection algorithms, such as model-based clustering [13, 16], COPA [9], and SIBER [17], on RNA-seq datasets, revealing widespread bimodal gene expression in development and disease [18]. Bimodal expression reflects critical biological dichotomies that underlie tissue differentiation, stem cell fate, and cancer heterogeneity [16, 19]. Furthermore, bimodal distributions have been associated with the origins of tumor diversity, drug resistance, and metastatic behavior [13, 20–24]. Understanding these patterns is thus vital for targeted therapeutic intervention and precision medicine.

However, these RNA-seq-centric approaches [13, 17, 25, 26] are fundamentally limited by the imperfect correspondence between RNA and protein levels—a relationship undermined by factors such as translation efficiency, post-translational modification, and protein stability. Notably, the correlation between transcript and protein abundance can be as low as 40%, diminishing the accuracy of RNA-seq-derived biomarkers in reflecting true functional heterogeneity [27, 28]. Proteins, as the direct effectors of cellular function, are more intimately tied to disease phenotypes than their RNA precursors. Proteomics, therefore, has the unique capacity to capture biologically relevant subgroups that may be invisible at the transcriptomic level, offering a fundamentally improved modality for biomarker discovery. By revealing bimodal protein expression, proteomics enables the identification of patient strata defined by actual biochemical activity, potentially yielding biomarkers with greater clinical utility and interpretability. A second major challenge in previous studies is the difficulty of distinguishing true bimodal expression from technical artifacts or insufficient sample sizes; rigorous models and larger datasets are thus required to reliably detect biologically meaningful bimodality [16, 17]. These limitations underscore the need to develop an appropriate bimodality model that can reliably identify bimodal expression patterns in proteomics datasets.

**Fig. 1:**
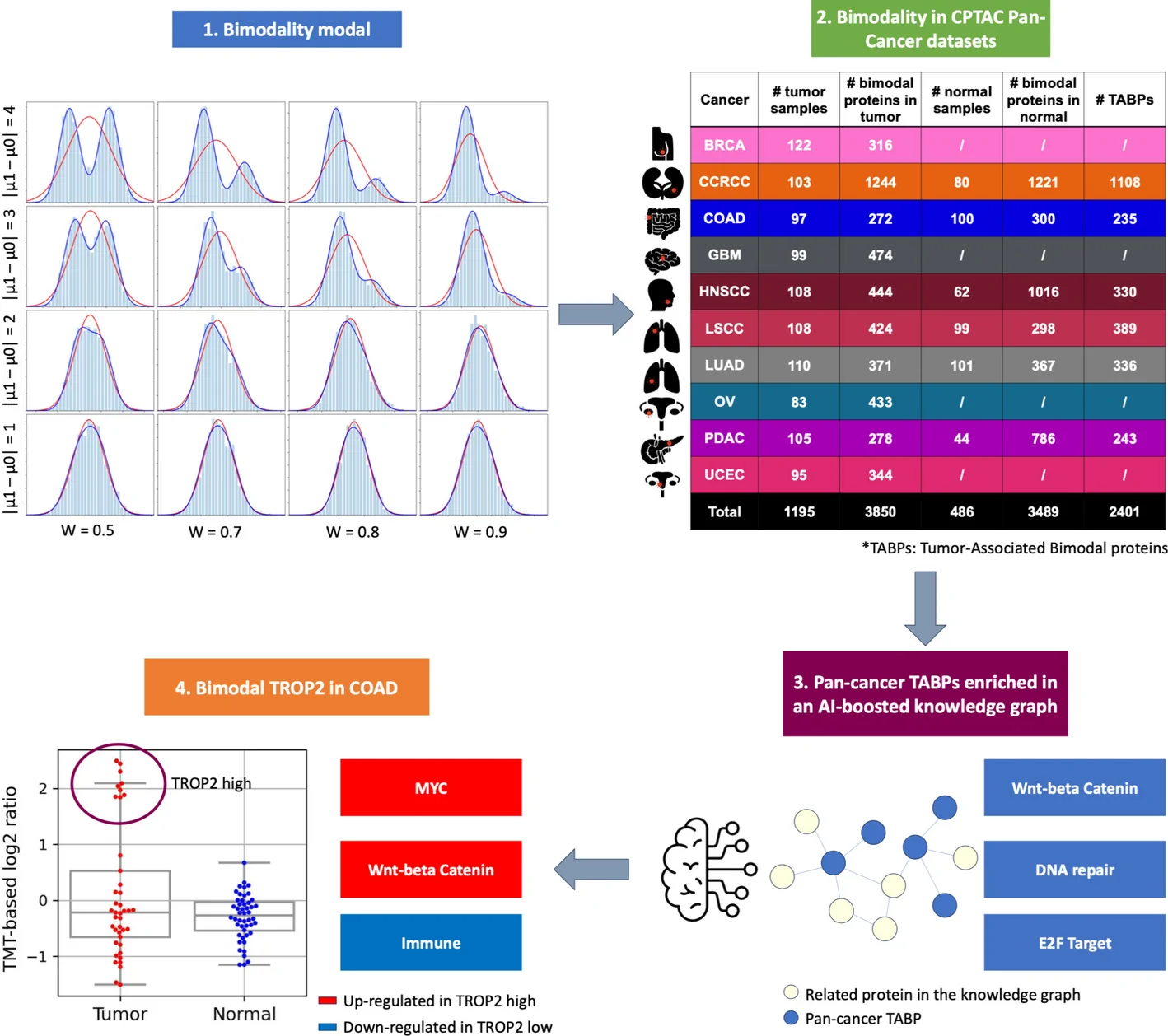
Workflow to (1) construct a bimodality model, (2) identify bimodal protein expressions in CPTAC pan-cancer proteomics data comprising 10 tumor cohorts and 6 normal cohorts, (3) capture the common patterns of pan-cancer tumor-specific bimodal proteins using an AI-based knowledge graph, and (4) explore biology behind *TROP2* bimodality in the COAD cohort

Recent advances in proteogenomics have paved the way to investigate cancer biology at unprecedented depth. The National Cancer Institute’s Clinical Proteomic Tumor Analysis Consortium (CPTAC) has generated comprehensive mass spectrometry–based proteomics datasets from large collections of tumors and matched normal tissues [3, 10, 29–40], establishing an ideal platform for pan-cancer bimodality analysis. In this study, we developed a robust bimodality model to systematically identify bimodally expressed proteins in CPTAC pan-cancer datasets [41], resulting in the discovery of 2,401 tumor-associated bimodal proteins (TABPs) (Fig. 1). By harnessing an AI-boosted knowledge graph [42], we revealed shared biological functions among 16 pan-cancer TABPs and conducted an in-depth case study of *TROP2*—a bimodal protein in colon adenocarcinoma—to dissect its mechanistic role and disease relevance. These findings demonstrate that bimodality in proteomics constitutes a powerful new modality for stratifying patients into biologically and clinically meaningful subgroups, uncovering unique biomarker hypotheses that complement and exceed what is possible with RNA-seq alone. The discovery of functional subgroups through proteomic bimodality represents a significant advancement, opening new avenues for pursuing precision oncology and individualized therapy.

## Results

### Development of a bimodal model for proteomics data

To comprehensively understand the scope of bimodal protein expressions, we implemented a bimodal model for proteomics data (Fig. 1). We first calculated three different bimodality metrics, (1) the bimodality index (*BI*), (2) the difference in Bayesian information criteria (*BIC diff*), and (3) kurtosis (*kurt*), on the simulation data sets (sample number *N* = 3000 or 100) with different combinations of mean differences and weights (Methods; Additional file 1: Fig. S1).

Additional file 1: Fig. S1A demonstrates a set of simulation data sets showing that, with large mean differences relative to variances and a sufficient sample size (*N* = 3000), bimodal distributions are easily identifiable. In such cases, *BI* exceeded 1, *BIC diff* ranged from 0 to –1100, and *kurt* varied between –1.3 and 0. In contrast, indistinguishable cases featured *BI* < 1, *BIC diff* 0–50, and *kurt* approximately 0. Increasing separation in the first column led to rising *BI* and decreasing *BIC diff* and *kurt*. We observed that positive *kurt* typically indicated outliers, whereas negative *kurt* suggested evenly distributed mass clusters. Given that imbalanced bimodality, which is common in real-world data, often resulted in *kurt* near 0, we excluded *kurt* from our bimodality model to avoid misinterpretations.

A major challenge in bimodality identification is limited sample sizes, which may be caused by experimental design or missing values. Additional file 1: Fig. S1B illustrates this challenge with a set of simulation data sets with only 100 data points (*N* = 100). Reduction of sampling from 3000 to 100 made it more difficult to discern cases previously categorized as easy. Despite smaller sample sizes, *BI* remained above 1 and *BIC diff* ranged from –38 to –16, indicating that these metrics can still effectively identify bimodal distributions under constrained conditions.

Next, we systematically evaluated *BI* and *BIC diff* using a benchmarking data set of 100 known bimodal and unimodal distributions (50:50; Methods, Additional file 1: Fig. S2). We sampled different sizes from the benchmarking data set (sample number *N* = 1000, 300, 100, 70, 50, 30) and separately computed the true-positive rate (TPR) and false-positive rate (FPR) at different thresholds for *BI* and *BIC diff* (Additional file 2: Tables S1–S2, Additional file 1: Fig. S3). To determine the optimal decision boundaries, we selected the threshold that maximized the Youden index (TPR – FPR) for each sample size. Notably, this empirical optimization indicated that the most effective cutoffs for distinguishing bimodality in this context sometimes extended to small positive values (e.g., < 3, < 4), differing from the strict theoretical boundary of < 0. At these empirically derived thresholds, *BIC diff* showed slightly higher TPRs and lower FPRs than BI at all sample sizes (Additional file 2: Tables S1–S2, Additional file 1: Fig. S3). To enforce stricter standards on FPRs, we decided to use both *BI* and *BIC diff* metrics and apply different thresholds according to the actual number of samples with protein quantifications in the proteomics data sets (Table 1).

**Table 1:** Bimodality thresholds for proteomics data sets with different sample sizes

*N*	*BIC diff* threshold	*BI* threshold
30–50	≤ 3	≥ 1.35
50–100	≤ 4	≥ 1.25
≥ 100	≤ 5	≥ 1.1

To ensure robust bimodality detection, we applied a filtering criterion requiring proteins to be quantified in at least 30 samples (n > = 30) with a Bimodality Index (BI) > = 1.1. We validated these thresholds via a comprehensive power analysis testing 15 scenarios across varying mixing weights and separation strengths (Additional file 3: Table S27). These simulations indicate that our approach prioritizes specificity; for distributions exhibiting strong bimodality (BI > = 2.0), a sample size of n = 30 yields approximately 70% detection power in conservative balanced scenarios (50/50 weights) and > 95% power in skewed scenarios (90/10 weights). Consequently, while we may under-detect proteins with weak bimodality (BI < 1.5), the candidates identified are highly likely to represent true bimodal distributions. Furthermore, to assess if missing data patterns could generate spurious results, we evaluated the impact of left-censored (truncated normal) missingness, which simulates mass spectrometry limits of detection. Since our mixture modeling relies strictly on observed values without imputation, left censoring generally reduces the likelihood of detecting bimodality by truncating the lower mode, rather than creating artificial separation. This confirms that the bimodal distributions reported here are robust to missingness artifacts.

### Prevalence of bimodality in CPTAC pan-cancer proteomics data

From the large-scale proteomics data of the CPTAC pan-cancer data sets, we analyzed 10 tumor cohorts: breast cancer (BRCA) [3], clear-cell renal cell carcinoma (ccRCC) [30], colon and rectal cancer (COAD) [43], glioblastoma (GBM) [38], head and neck squamous-cell carcinoma (HNSCC) [44], lung squamous-cell carcinoma (LSCC) [45], lung adenocarcinoma (LUAD) [46], ovarian cancer (OV) [39], pancreatic ductal adenocarcinoma (PDAC) [29], and uterine corpus endometrial carcinoma (UCEC) [47]. We identified bimodal proteins in the 10 tumor cohorts as well as in the 6 matched normal cohorts (ccRCC, COAD, HNSCC, LUAD, LSCC, and PDAC) using our bimodality model (Methods; Fig. 1, Additional file 2: Tables S4–S19).

**Fig. 2:**
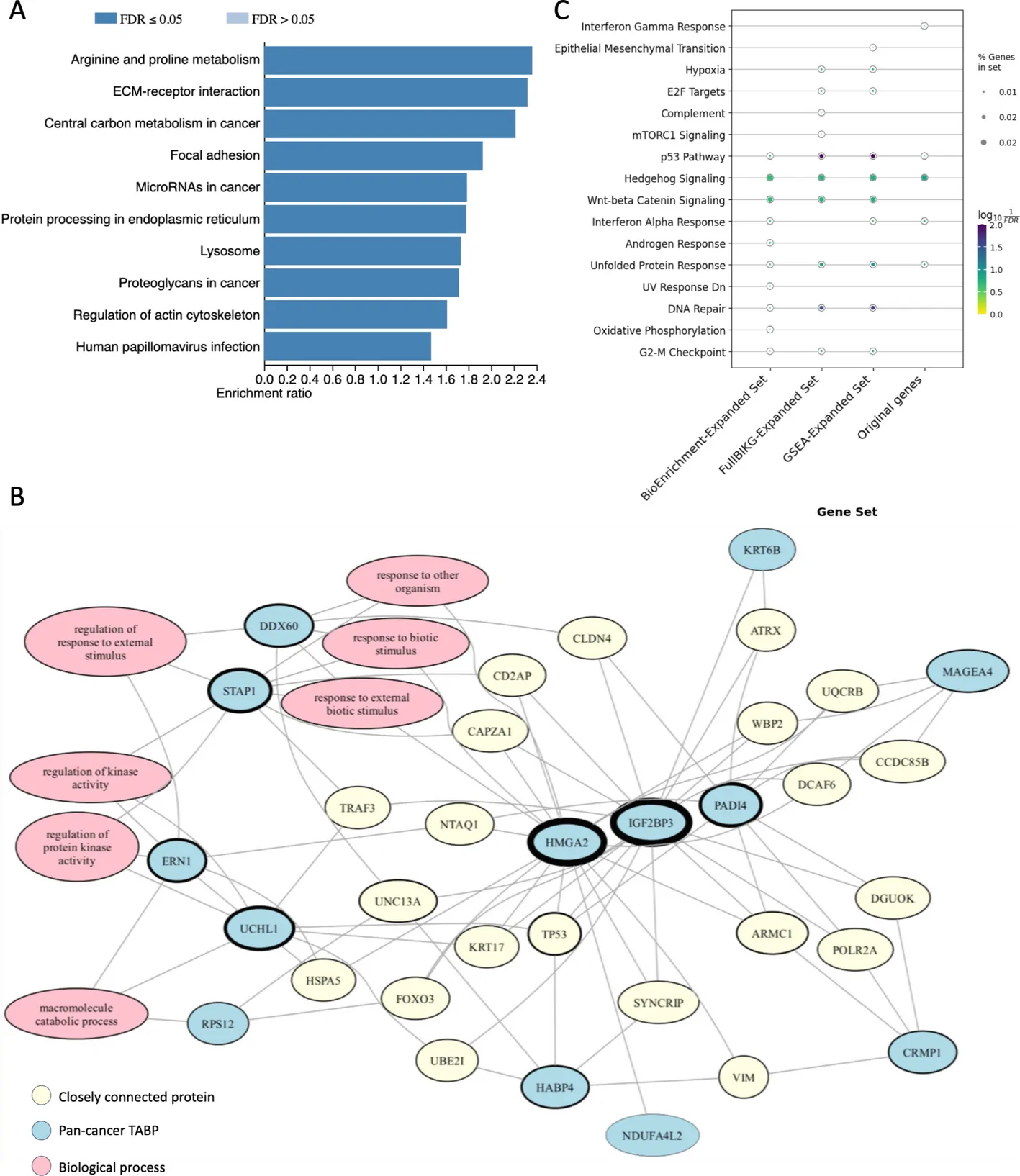
Signaling pathways associated with the TABPs. **A** Enrichment analysis of the tumor-associated bimodal proteins. **B** The subnetwork extracted from BIKG with 16 pan-cancer TABPs as seed nodes of PageRank. Blue nodes indicate the original TABPs; yellow, closely connected proteins to the TABPs; pink, biological processes. **C** Enrichment analysis using the BIKG-enriched proteins and original TABPs. The sizes of the colored circles represent the percentage of the gene covered in the gene set; colors represent –log10(FDR)

To focus our study on tumor-associated bimodality, we defined TABPs as bimodal proteins that are found in the tumors but not present in the matched normal tissues (2401 of 15,098 proteins; Methods, Additional file 2: Table S3). Pathway enrichment analysis revealed a significant association of TABPs with arginine and proline metabolism, extracellular matrix (ECM)-receptor interaction, central carbon metabolism in cancer, focal adhesion, and protein processing in endoplasmic reticulum in different cancer types (Fig. 2A, Additional file 3: Table S20). For example, 15 ccRCC-specific TABPs (*CD47*, *COL6A3*, *FN1*, *HSPG2*, *ITGA11*, *ITGAV*, *ITGB4*, *ITGB7*, *LAMA2*, *LAMB3*, *LAMC2*, *NPNT*, *THBS1*, *VTN*, *VWF*; Additional file 1: Fig. S4A) overlapped with the ECM-receptor interaction gene set, which primarily consists of various fibrillar collagens, fibronectin, and basement membrane components like laminin, collagen IV, and heparan sulfate proteoglycan [48]. The bidirectional interaction between cells and the ECM influences cell adhesion and migration [49].

To understand the functional characteristics of TABPs, we performed enrichment analysis comparing 2,401 TABPs against the complete CPTAC proteome background (15,098 proteins measured across 10 cancer types) for housekeeping genes, structural genes, oncogenes, and tumor suppressors using Fisher's exact test (Additional file 3: Table S25). TABPs were significantly enriched for structural genes (49 genes, 2.05-fold enrichment, *p* = 2.84 × 10⁻⁷) including ribosomal proteins, core histones, cytoskeletal components (tubulins, actins, keratins), extracellular matrix proteins (collagens, fibronectin, laminins), and proteasome subunits. TABPs were also enriched for housekeeping genes (46 genes, 1.61-fold enrichment, *p* = 5.02 × 10⁻^4^) encompassing essential metabolic enzymes, protein synthesis machinery, and ATP synthase components. In contrast, TABPs showed no significant enrichment for oncogenes (24 genes, 1.20-fold, *p* = 0.19) or tumor suppressors (16 genes, 0.78-fold, *p* = 0.89). These findings indicate that bimodal protein expression in tumors preferentially affects genes involved in basic cellular architecture and metabolism rather than canonical cancer driver genes, suggesting that TABPs primarily reflect intratumor heterogeneity in cellular states, metabolic activity, and structural organization rather than heterogeneity in oncogenic mutations.

Next, we investigated 16 pan-cancer TABPs (*IGF2BP3*, *NDUFA4L2*, *EPS8L3*, *PADI4*, *UCHL1*, *TLNRD1*, *MSANTD2*, *CRMP1*, *ERN1*, *STAP1*, *RPS12*, *HABP4*, *KRT6B*, *MAGEA4*, *DDX60*, *HMGA2*) that were exhibited bimodal distributions in at least three different cancer indications (Additional file 1: Fig. S4B, Additional file 3: Table S21). We reasoned that these 16 pan-cancer TABPs and their related pathways might reveal potential biomarkers. We utilized an AI-boosted knowledge graph, the Biological Insight Knowledge Graph (BIKG) [42], and network propagation with the pan-cancer TABPs as seed nodes (Methods). In the BIKG-expanded network, we clustered 13 pan-cancer TABPs and 21 closely connected proteins (Fig. 2B). Of the 21 proteins, 5 (*KRT17*, *TP53*, *TRAF3*, *UBE2I*, *UNC13A*) were identified as bimodal proteins in at least one tumor cohort in the CPTAC pan-cancer data sets (Additional file 1: Fig. S4C–I), indicating that the AI-based knowledge graph could capture common signals on pan-cancer TABPs (chi-square test, *P* < 0.0001). Pathway enrichment analysis revealed a significant association of the proteins in the network with *E2F* targets, p53 pathway, *WNT*/β-catenin signaling, and DNA repair that were interconnected and played an important role in the development and progression of cancer [50–53] (Fig. 2C).

### *TROP2* bimodality in COAD

*TROP2*, also known as tumor-associated calcium signal transducer 2 (*TACSTD2*), is a calcium signal transducer that is often overexpressed in many solid tumors, including colorectal cancer [54, 55]. Increased *TROP2 *expression is associated with tumor aggressiveness, epithelial-to-mesenchymal transition, metastasis, and decreased overall survival [54, 56, 57]. These characteristics have triggered an interest in developing *TROP2*-targeted therapies for patients with colorectal cancer [58]. In our study, *TROP2* was identified as one of the top TABPs in COAD, leading us to further explore *TROP2* bimodality to understand the mechanisms underlying this biological status.

**Fig. 3:**
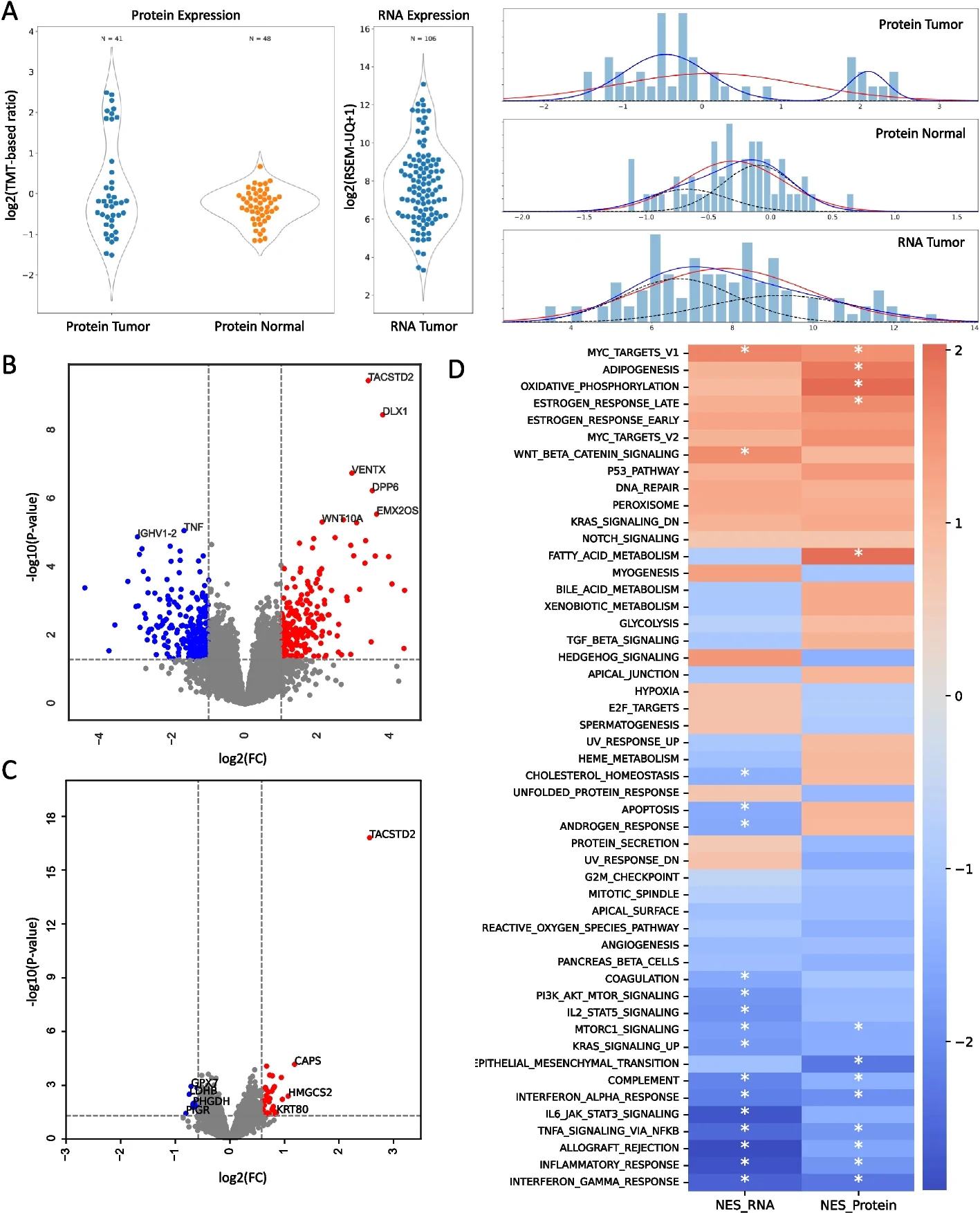
*TROP2* (*TACSTD2*) bimodality in the CPTAC COAD. **A** Distributions of *TROP2* protein expression in CPTAC COAD tumor and normal samples and RNA expression in tumors. Bimodality was identified only in proteomics data in tumor samples. The red line indicates the corresponding unimodal fit, and the blue and black lines show the bimodal fit. **B**, **C** Volcano plot showing differentially expressed mRNA and proteins (panels **B** and **C**, respectively) between *TROP2*-high and *TROP2*-low samples in the CPTAC COAD cohort. Red indicates significantly upregulated genes (*P* < 0.05, log(FC) > 0.58); blue, significantly downregulated genes (*P* < 0.05, log(FC) < –0.58). **D** Heat map showing results of GSEA. Normalized enrichment scores are plotted for hallmark pathways from MSigDB based on differentially expressed genes and proteins (statistically significant results are indicated by an asterisk)

*TROP2* (*TACSTD2*) was reliably quantified in 41 tumor samples in the COAD cohort and had a bimodal distribution in tumor samples (*BI*, 3.24; empirical *p*-value, 0.003; 95% confidence interval [CI], 1.74–3.96; *BIC diff*, −24.08; empirical *p*-value, 0.0001; 95% confidence interval [CI], −42.19 to −11.18; Fig. 3A) but not adjacent normal colon tissues. Statistical analysis of RNA-seq data for the *TROP2* mRNA transcript (*BI*, 0.76; empirical *p*-value, 0.7; 95% confidence interval [CI], 0.55 to 2.10; *BIC diff*, 10.30; empirical *p*-value, 0.2; 95% confidence interval [CI], −13.51 to 14.10) did not support (*p*-values of rejecting null hypothesis of unimodality 0.7 and 0.2 from *BI* and *BIC diff*, respectively) the presence of a bimodal distribution in COAD tumors at the transcriptomic level (Fig. 3A). Samples were classified as *TROP2*-high or *TROP2*-low by fitting a two-component Gaussian Mixture Model (GMM) to the *TACSTD2* protein expression values and assigning each sample to its most probable component via posterior probability. The GMM-derived decision boundary (1.34) fell within a natural gap in the data (0.80–1.84), yielding 9 high and 32 low samples. *TACSTD2* mRNA levels were also significantly higher for the *TROP2*-high samples than for the *TROP2*-low samples (Mann–Whitney U-test, *P* ≤ 10e-4; Additional file 1: Fig. S5A), in agreement with the strong correlation between *TROP2* protein and mRNA levels (Spearman correlation, *P* = 1.5e-15; Additional file 1: Fig. S5B). To further validate the discovery of *TROP2* bimodality in colorectal cancer, we explored two independent proteomics data sets, from the T1NxM0 colorectal cancer (CRC) cohort in Chinese population and The Cancer Genome Atlas (TCGA) colorectal cancer cohort, and showed that in both datasets, *TROP2* had bimodal distributions in tumor samples (T1NxM0 CRC cohort [59]: *BI* = 3.10, 95% confidence interval [CI] = 0.71–9.05, *BIC diff* = –3.00, 95% CI = –44.5 to 9.0; TCGA CRC cohort [43]: *BI* = 1.41, 95% CI = 0.52–4.10, *BIC diff* = –66.6, 95% CI = –108.2 to –34.8; Additional file 1: Fig. S5C, D).

Next, we showed that *TROP2* bimodality is not confounded by demographic and clinical features, chromosomal instability phenotype (CIN), microsatellite instability phenotype (MSI), or transcriptomic subtype (Additional file 1: Figs. S6, S5E, F). Multi-omics factor analysis (MOFA) [60] identified other features associated with *TROP2* abundance. One of the latent factors fitted by the model (identified post-hoc) had a high-weight contribution from both the *TROP2* protein and mRNA abundance features but also from multiple copy number alteration features. Fisher exact test revealed that samples classified as *TROP2*-high were enriched for copy number amplifications of top genes associated with this latent factor (*P* = 0.006; Additional file 1: Fig. S5G, Additional file 3: Table S24). *MYC* was identified as one of the top 10 genes associated with this latent factor through MOFA analysis and was a hub node in STRINGdb, connecting with 54 copy number alteration genes, including *VEGFR* and *CCNE1*/*CCNE2*. *VEGFR*, whose promoter is known to be targeted by *MYC*, is implicated in angiogenesis, while *CCNE1* and *CCNE2* play essential roles in G1/S cell cycle progression. However, statistical tests revealed that *MYC* copy number amplification was nonsignificant (*P* = 0.06; Additional file 1: Fig. S5H), and *MYC* mRNA expression showed no significant difference between *TROP2*-high and *TROP2*-low samples (*P* = 0.74; Additional file 1: Fig. S5I).

### Therapeutic opportunities driven by *TROP2* bimodality

Differential RNA-seq/protein expression analysis between *TROP2*-high and *TROP2*-low samples using the DESeq2 package revealed a number of upregulated and downregulated genes/proteins (Fig. 3B, C; Additional file 3: Tables S22–S23). Pathway enrichment for the differentially expressed genes and proteins revealed concordant enrichment of hallmark pathways, the most notable upregulated pathways in the *TROP2*-high group being the *MYC* pathway and *WNT*/β-catenin signaling pathways consistent with previous literature implicating *TROP2 *in cell proliferation, apoptosis, and cell growth [61–63]. In the *TROP2*-low group, inflammatory and interferon-related pathways, as well as tumor necrosis factor-alpha and *IL6*-JAK-*STAT3* signaling pathways, were upregulated (Fig. 3D).

**Fig. 4:**
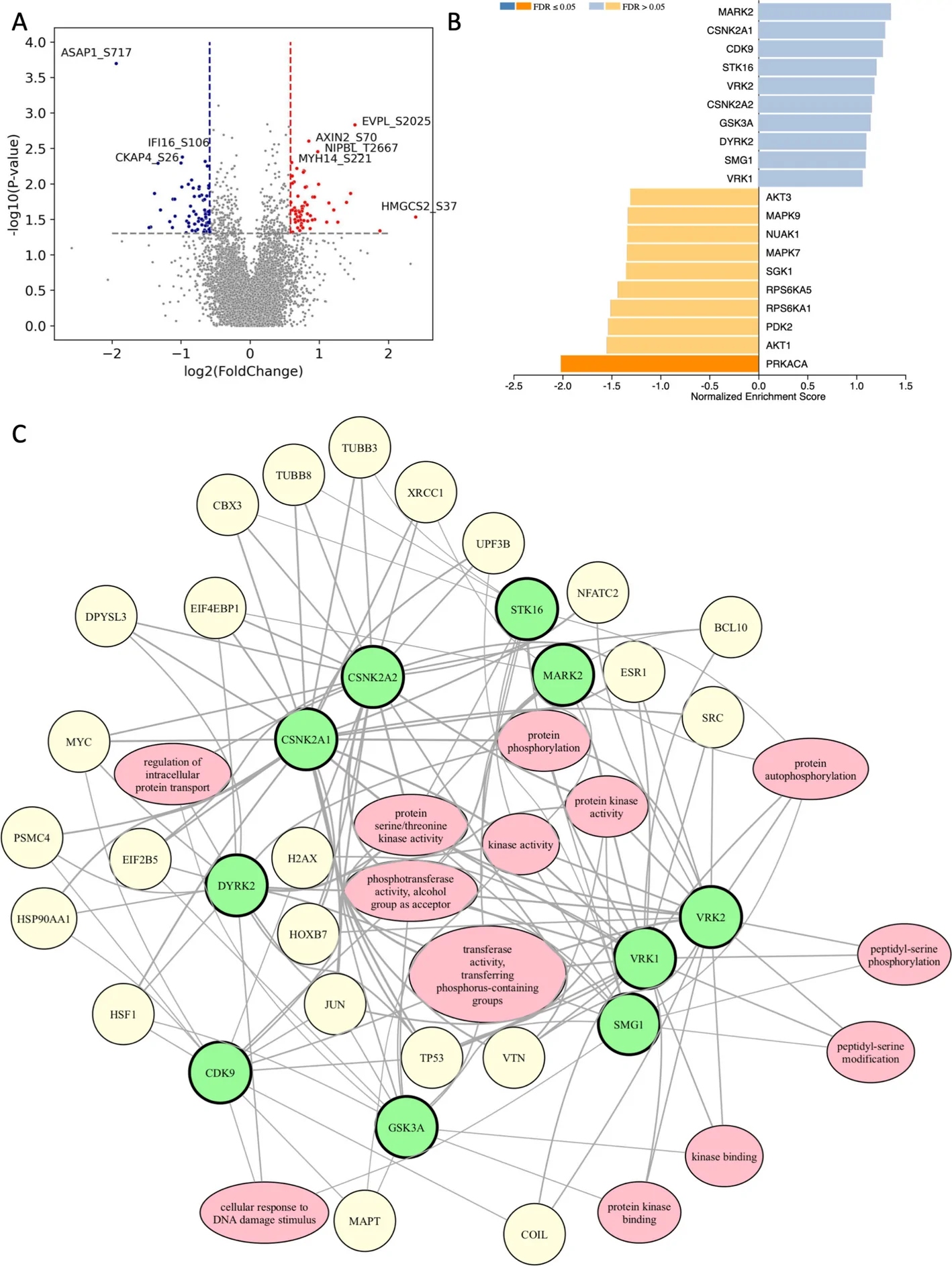
*TROP2* bimodality and immune suppression in COAD. **A** Volcano plot showing differentially expressed phosphosites between *TROP2*-high and *TROP2*-low samples in the CPTAC COAD cohort. Red indicates significantly upregulated phosphosites (*P* < 0.05, LFC > 0.58); blue, significantly downregulated phosphosites (*P* < 0.05, LFC < –0.58). **B** Kinase activity inferred from differentially expressed phosphosites using GSEA. **C** Network extracted from BIKG with upregulated kinases as seeds. Green nodes indicate the upregulated kinases; yellow, closely connected proteins to the upregulated kinases; pink, biological processes

Phosphoproteomics analysis showed that in the *TROP2*-high samples, the most significantly upregulated phosphosites were EVPL_S2025, AXIN2_S70, NIPBL_T2667, and MYH14_S221 (Fig. 4A). Kinase activity inference from phosphoproteomics identified some upregulated kinase activities in *TROP2*-high samples (false discovery rate, FDR > 0.05; Fig. 4B). We utilized those kinases as seed nodes to explore a corresponding network in BIKG [42] (Methods; Fig. 4C), highlighting potentially associated proteins and biological processes. Pathway analysis based on this network revealed a correlation between the upregulated kinases and *WNT*/β-catenin signaling (Additional file 1: Fig. S7A), consistent with prior differential analyses using proteomics/RNA-seq data.

In contrast, kinase activity inference suggested that *TROP2*-low samples had significantly higher *PRKACA* kinase activity (FDR < 0.05; Fig. 4B). *PRKACA* protein expression was significantly correlated with the *PI3K*-*AKT*-*MTOR* signaling pathway in COAD (Additional file 1: Fig. S7B). From BIKG-based network analysis, we identified upregulated immune-related pathways (Additional file 1: Fig. S7C, D), which were also observed in the previous mRNA/protein DE analyses. Using the immune scores from ESTIMATE [64], we further observed that *TROP2*-low samples had more activated immune signaling than *TROP2*-high samples (Additional file 1: Fig. S7E). The immune subtypes from a previous CPTAC study [65] showed that *TROP2*-low samples are enriched with CD8-/interferon gamma-positive (*IFNG*^+^) subtypes as compared with CD8^–^/*IFNG*^–^ subtypes (chi-square test, *P* = 0.005; Additional file 1: Fig. S7F). CD8^–^/*IFNG*^+^ represents an immune subtype with low immune infiltration of CD8 T cells and B cells, but strong activation of IFN signaling. CD8^–^/*IFNG*^–^ was low in both functions (immune cold). While these multi-omics associations provide a biological rationale for potential therapeutic stratification, further investigation in clinical cohorts is required to validate these hypotheses.

## Discussion

We developed a bimodal model tailored for patient-derived proteomics data, which are often characterized by smaller sample sizes and more missing values than RNA-seq data. We used simulated bimodal and unimodal distributions across various sample sizes to provide reliable benchmark data for evaluating bimodality metrics. Theoretically, the *BI* and *BIC diff* metrics are very similar, in that they are derived from the same fitting procedure. A negative *BIC diff* indicates a bimodal preference, whereas a positive value suggests a unimodal fit. Unlike *BIC diff*, *BI *does not have defined thresholds and usually uses percentile thresholds or heuristic thresholds [13, 16, 19, 20]. Our results also indicated that smaller sample sizes affect the sensitivity and specificity of both metrics. We validated and established optimal thresholds for *BI* and *BIC diff* using a benchmarking data set that reflected actual sample conditions in proteomics data. This rigorous methodology ensured the robustness and reliability of our bimodality model, even under constrained sample conditions.

Our investigation into the bimodality of protein expressions within the CPTAC pan-cancer proteomics data has revealed insights into the molecular underpinnings of cancer biology. The identification of 2401 TABPs underscores the prevalence of bimodal expression in cancer and its potential utility in refining diagnostic and therapeutic strategies. Notably, our approach leverages advances in proteogenomics, addressing previous limitations inherent in RNA-based studies by directly incorporating protein expression data. This shift is crucial, given the often modest correlation between RNA and protein levels, which may not adequately reflect post-transcriptional modifications and the true biological state of the cells. For example, in contrast to findings in a previous pan-cancer RNA-seq–based bimodality study, we identified 2253 unique TABPs, of which 148 were overlapping [20].

Our findings highlight the significant associations of TABPs with critical cancer pathways, such as arginine and proline metabolism, ECM-receptor interaction, and focal adhesion. These pathways are integral to tumor progression and metastasis, suggesting that proteins with bimodal expression patterns could serve as candidate biomarkers for these biological processes in various tumor types. Moreover, the application of AI-boosted knowledge graphs in our analysis allowed for the identification of common patterns among these proteins across different cancers, potentially offering new avenues for targeted therapies.

*TROP2 *has been previously described as both a prognostic biomarker and a therapeutic target [62]. Interestingly, our study identified two patient populations characterized by bimodal *TROP2* expression. An in-depth multi-omics analysis revealed distinct disease biology associated with these populations, suggesting that *TROP2* may serve as a novel biomarker for colon cancer. Specifically, MOFA analysis indicated that the *TROP2*-high group was strongly associated with the *MYC* signaling pathway, as revealed through differential RNA and protein pathway enrichment analysis. Despite this finding, statistical tests showed that *MYC* was not significantly copy number-amplified (*P* = 0.06; Additional file 1: Fig. S5H), and its mRNA abundance did not differ between *TROP2*-high and *TROP2*-low samples (*P* = 0.74; Additional file 1: Fig. S5I). This finding suggests that *MYC* pathway activation may occur through indirect mechanisms, potentially involving alternative regulatory elements or post-translational modifications.

In addition, RNA and protein analyses identified enrichment of the *WNT*/β-catenin pathway in the *TROP2*-high population. Previous studies have shown that *TROP2* regulates self-renewal, proliferation, and transformation through regulated intramembrane proteolysis (RIP), which cleaves *TROP2 *into extracellular and intracellular domains [62, 66]. *TROP2* can enhance the self-renewal of stem cells and progenitor cells via β-catenin signaling and can facilitate the development of precursor lesions in prostate cancer in vivo, with disruptions occurring in the absence of β-catenin or *TROP2 *RIP cleavage mutants [62, 66–70]. Our findings suggest that *TROP2 *may have an analogous role in colon cancers. The Colorectal Cancer Subtyping Consortium (CRCSC), a group that analyzed gene expression data in more than 30 expression sets spanning multiple platforms and sample preparation methods, has proposed consensus molecular subtypes (CMSs) based on genome-wide gene expression profiling analysis, which categorizes colorectal cancers into four groups (CMS1–4) [71, 72]. *TROP2*-high colorectal cancer tumors are enriched in the CMS2 subtype of colorectal cancer, which is also characterized by *WNT*/β-catenin activation [72]. Furthermore, phosphoproteomics analysis revealed a significant increase in *AXIN2* phosphorylation. As an inhibitor of the *WNT*/β-catenin pathway, *AXIN2* facilitates the phosphorylation of β-catenin and adenomatous polyposis of the colon by *GSK3B* [73, 74] and promotes the degradation of β-catenin [75]. This phenomenon may represent a negative feedback loop through *AXIN2* to control β-catenin activation in colorectal cancer, potentially driven by *TROP2 *expression [73].

In contrast, *TROP2*-low populations are distinguished by activated *PI3K*-*AKT* pathways and inflammatory and interferon-related pathways, as demonstrated by differential RNA and protein expression pathway enrichment analysis. Phosphoproteomics analysis showed that *TROP2*-low samples had significantly higher *PRKACA* kinase activity (FDR < 0.05; Fig. 4B). *PRKACA* protein expression significantly correlated with the *PI3K*-*AKT*-*MTOR* signaling pathway in COAD (Additional file 1: Fig. S7B), suggesting that in *TROP2*-low samples, *PRKACA* could drive colorectal cancer progression via the PKA-*FAK*-*AKT* pathway.

These findings suggest that oncogenesis and tumor proliferation in the two populations are potentially driven by distinct signaling pathways and possess different tumor microenvironments. Therefore, it is crucial to consider therapeutic interventions guided by *TROP2* protein expression as a biomarker. This personalized approach could enhance treatment efficacy by targeting the specific molecular drivers present in each tumor subtype. For instance, if the role of *TROP2* in self-renewal and transformation is validated in the *TROP2*-high colorectal cancer population, therapeutics that block *TROP2* activation might constitute an effective treatment strategy for this group. Further experiments are warranted to test this compelling hypothesis.

Despite these advancements, our study has limitations that must be acknowledged. Most significantly, any functional claims regarding potential therapeutic targets are based on computational associations and literature synthesis rather than direct experimental evidence. While we identified robust correlations between bimodal protein expression and specific oncogenic pathways, we did not perform mechanistic experiments in vitro or in vivo to validate the functional consequences of these expression patterns. Consequently, the biological impact and therapeutic viability of the identified TABPs remain to be confirmed in wet-lab settings. Additionally, our study relied on available proteomics data that does not include all cancer types or all stages of disease progression. CPTAC has insufficient therapeutic information to uncover biomarkers with our methods. In addition, although we demonstrated the application of our bimodality model to a large data set, the generalizability of these findings to other data sets or through prospective validation remains to be tested. One example we showed is *TROP2* bimodality validation in two external data sets.

Future research should prioritize expanding the application of bimodality analyses to other types of cancer and integrating multi-omics data to further validate and refine the identified biomarkers. Crucially, follow-up studies must include mechanistic experiments to substantiate the functional roles of high-priority TABPs suggested by our model. Moreover, clinical trials could explore the therapeutic implications of these findings, particularly the differential strategies suggested by *TROP2* bimodality in colorectal cancer.

## Conclusions

In conclusion, our study not only provides a robust framework for the identification of bimodal proteins expressed across cancers but also highlights the role of such biomarkers in elucidating the complex molecular landscape of malignancies, offering promising prospects for precision oncology.

## Methods

### Simulated bimodality distributions

Data simulation was carried out by creating linear combinations of two normal distributions. The number of bimodal genes was controlled by sampling a binomial distribution. For the genes chosen as bimodal, a weight was randomly sampled. The difference in means between the modes of the normals was randomly sampled from a uniform distribution, assigning the value to the first mode and 0 to the second. If the genes were selected as unimodal, then both means were assigned as equal. Finally, the variance was kept equal for both modes and randomly chosen from a uniform distribution. See “Benchmarking BI and BIC diff” below for mode details.

### Bimodality metrics

The approach used to detect proteins with bimodal expression entailed determining whether the distribution is best described as unimodal or bimodal. Technically, the approach involved either (1) fitting the data to these two models and using goodness-of-fit to make a choice or (2) using a metric that transitions to different values if the data are unimodal or bimodal. Both the *BI* and the *BIC diff* belong to the first class, whereas measuring *kurt* belongs to the second one. For each simulation data, the *kurt* of the data was computed directly from the samples. The *BI* and the *BIC diff* were computed after fitting a normal (unimodal) or a mixture of two normal (bimodal) distributions, indicated by the respective red and blue lines in Additional file 1: Fig. S1.

To detect proteins or genes with bimodal expression, three different metrics were computed (Additional file 2: Tables S1–S2). The first metric was used to obtain the *kurt* of the distribution that measures the “tailness,” by computing:

$$k= \frac{\frac{1}{n}{\sum}_{i=1}^{n}{\left({x}_{i}-\overline{x }\right)}^{4}}{{\left(\frac{1}{n}{\sum}_{i=1}^{n}{\left({x}_{i}-\overline{x }\right)}^{2}\right)}^{2}}-3$$

The second metric was used to determine the difference in goodness-of-fit (measured by the *BIC*) when the data were fitted using a normal distribution or a mixture of normal distributions, by computing:

BIC=klog(n)-2log(likelihood)

BICdiff=BIC(bimodal)-BIC(unimodal),

where *likelihood* is the maximized value of the likelihood for the model, *n* is the number of data points, *k* is the number of parameters, *BIC*(*bimodal*) is the score obtained upon fitting a weighted mixture of normal distributions, and *BIC*(*unimodal*) is the score obtained by fitting a normal distribution. Negative values suggest that the data are better modeled as a mixture of normals, whereas positive values indicate a unimodal distribution.

The third metric reused the models fitted above and computed the *BI*:

$$BI =sqrt(w1(1- w1)) abs(mu1- mu2) / sqrt((1-w1) * sigma1^2 + w1 * sigma2^2),$$

where *w1*, *mu1*, *sigma1*, *mu2*, and *var2* are the bimodal distribution fitted:

$$y = w1 \times N(mu1, sigma1^2) + (1- w1) \times N(mu2, sigma2^2)$$

and *N*(*mu1*, *sigma1*^2^) is Gaussian. In general, *BI* is higher for distribution showing bimodality. This can be observed directly in the formula above. If the weights for the two modes are similar, w1*(1 – *w1*) is higher. If the separation for the modes is high, then abs(*mu1* – *mu2*) is higher. If the spread of each mode is lower, then the denominator is smaller, and *BI* is larger.

One potential challenge posed by this type of analysis is how to assess the confidence of the metrics when the number of points used to fit the distribution is small. To overcome this issue, the samples for each protein analyzed were bootstrapped 300 times and the corresponding 95% CIs were computed.

### Benchmarking *BI* and *BIC diff*

*BI* and *BIC diff* metrics are very similar, given that both arise from the same fitting procedure. To assess the performance of these metrics, we created a set of known bimodal and unimodal distributions (*N* = 100). The number of bimodal and unimodal distributions was randomly chosen from a binomial distribution with *P* = 0.5. To completely define each distribution, we randomly sampled the following parameters:

- W ~ uniform(0.5, 0.90)
- U1 ~ uniform(−4, −2.5)
- Var1 ~ uniform(0.5, 2)

If the distribution was unimodal, then we created data following N(U1, Var1); if the distribution was bimodal, we created data following w*N(u1, var1) + (1-w1)*N(0, var1).

### Bimodality identification using CPTAC pan-cancer proteomics data sets

Ten tumor cohorts and six normal cohorts in CPTAC pan-cancer data sets (with at least 40 samples in the proteomics data) were analyzed. For each cohort, we calculated *BI* and *BIC diff* for each protein that was quantified in at least 30 samples in the cohort (Additional file 2: Tables S4–S19). To define bimodal proteins, we selected proteins according to the thresholds in Table 1. TABPs were defined as those proteins identified as bimodal proteins in a tumor cohort but not in the matched normal tissues.

### Prioritizing proteins of interest using BIKG

Biological understanding of TABPs or differential proteins was identified using knowledge graph network analysis on the Biological Insight Knowledge Graph (BIKG)—an AI-augmented system constructed using natural language processing and machine learning to integrate relationships between diverse biomedical data [42]. Knowledge graph data structures provide flexibility in network modeling by providing variants of both edge and node types. In this study, we used this analysis for two cases: (1) expanding gene sets of interest to identify related gene targets and (2) using biologically relevant node types (genes, pathways, molecular functions, disease, and biological processes) to find underlying biology to describe the relationships between our target genes. The two knowledge graphs were constructed using data from databases including: METABASE, DOROTHEA, HETIONET, and CELLPHONEDB. In addition, relations between gene nodes in both subgraphs were filtered to contain edge relations of types: interacts, regulates, or modifies. Relations between gene targets and other node types in the biological enrichment graph were of the type “participates” for biological process, pathway, and molecular function node types, and “up/downregulates” for connections with disease nodes. For each gene set of interest, the two subgraphs were further filtered to retain edges connecting either genes with this list itself or connecting any node in this set to any other node in the graph. Such filtering provided a more manageable size of network to analyze.

A repeated random walk procedure was performed where *N* = 1000 random paths were traversed originating from each node in our target gene list. Upon performing all the walks, edges that had a number of occurrences above a certain threshold were retained. This random walk procedure was repeated until the obtained network did not change between iterations, providing network stability. By leveraging these algorithmic traversals, we moved beyond conventional database lookups to uncover "dark matter" connections—biologically relevant associations lacking direct literature support. Such subgraphs generated for each gene list of interest provided expanded gene signatures of interest as well as biological events that may relate to our genes. The expanded gene sets were then used in gene set enrichment analysis (GSEA) to identify additional pathways related to the larger gene set expanded using the knowledge graph approach. Given that BIKG includes proprietary data (METABASE) and integration methods, the insights generated through this process should be treated as hypothesis-generating.

### Transcriptomic subtypes in COAD

The transcriptomic subtype classification of each sample was performed according to the Consensus Molecular Subtypes framework as described in Guinney et al. [71].

### Microsatellite instability status and CIN in COAD

Both MSI status and CIN information was obtained from LinkedOmics according to detailed methods described by Vasaikar et al. [76, 77].

### Statistical hypothesis test

The chi-square test of independence was used to assess whether there was a significant association between the CMS transcriptomic subtypes/demographic/clinical features/MSI/CIN and *TROP2* high versus low status.

### Multifactor omics integration using MOFA + 

MOFA [78] is a latent variable model that relies on matrix factorization and Bayesian modeling to uncover orthogonal axes of heterogeneity in multimodal data sets. The dependencies between omics layers are modeled by decomposing data into a lower-dimensional representation of shared factors and data set–specific contributions. This approach enables the identification of molecular signatures that are concordant across different omics modalities while accounting for data set–specific variation. In our study, MOFA was employed to determine which omics features, including proteomics, transcriptomics, copy number variations, miRNAs, mutations, and phospho-proteomics, were associated with the bimodal *TROP2* protein distribution in the CPTAC COAD patient cohort. Features with the highest weight in the MOFA factor that also had a high-weight contribution from *TROP2* protein were selected for downstream investigation.

### Differential analysis (RNA-seq, proteomics, and phosphoproteomics)

Tumor samples from the COAD cohort for RNA-seq, proteomics, and phosphoproteomics were used for differential expression analysis between *TROP2*-high and *TROP2*-low subgroups.

For RNA-seq, differential expression analysis was performed using the PyDESeq2 [79, 80] (version 0.3.1) package in Python 3.9. Briefly, raw count data for 60,660 gene transcripts were loaded into Python, and low-count genes were filtered out to minimize noise (requiring that on average, each transcript has been detected 10 times in each sample), yielding a total analysis set of 19,419 reliably expressed transcripts. Normalization factors were calculated to account for differences in library size, and the negative binomial distribution was fitted to the count data. Dispersion estimation and Wald tests were performed to identify genes that were differentially expressed between the *TROP2*-high and *TROP2*-low samples. To correct for multiple hypothesis testing, *P *values were adjusted using the Benjamini–Hochberg method [81]. Genes with an adjusted *P* < 0.05 were considered to be significantly differentially expressed.

For proteomics, proteins that were quantified in at least 50% of the samples were used for further analysis. Imputation and differential protein expression analysis was performed using DEP package in the R version 4.3.1 environment as described in Wang et al. [41]. The fgsea package [82] was used for protein gene set enrichment analysis. The fgsea algorithm was run with 1000 permutations.

For phosphoproteomics, a phosphosite must have been quantified in at least 50% of tumor samples or was removed. The unpaired Wilcoxon rank-sum test was used to calculate significance. Significantly differential sites were defined as *P* < 0.05 and Fold Change, *FC* > 1.5.

### GSEA

The GSEA “prerank” method [83] was employed to assess pathway enrichment, using a list of genes ranked by *P* values from differential RNA/protein analysis or copy number variations associated with MOFA latent factors. GSEA was used to evaluate whether predefined gene sets (“hallmarks” or “C1 positional gene sets corresponding to human chromosome cytogenic bands”) from the MSigDB database were significantly enriched toward the top (positively enriched) or the bottom (negatively enriched) of the ranked gene/protein list. Statistical significance was determined through a permutation-based approach that computed enrichment scores and false discovery rates.

### Kinase-substrate association database

We constructed the gmt files for GSEA to infer kinase activity. The gmt file is the kinase substrate association database that combines the ground truth kinase substrate associations and the predicted kinase substrate associations from a machine learning-based tool. The ground truth kinase substrate associations were downloaded from GPS 5.0 [83], a curation of 15,194 experimentally identified kinase substrate associations. The predicted kinase substrate associations were obtained from CoPheeKSA [84]. When a kinase had more than 100 known substrates in the ground truth dataset, no predicted substrates were added to this kinase in the new kinase substrate association database. When a kinase had fewer than 100 known substrates in the ground truth dataset, the predicted substrates from CoPheeKSA were added to this kinase, with a maximum number of 500 (the phosphosites ranked by prediction scores from CoPheeKSA).

### Kinase activity inference

We used Webgestalt [85] to perform GSEA on the phosphosites and the –log10(*P*) from differential analysis [86]. The organism was set to “other.” The functional database was uploaded using a self-defined gmt file (kinase-substrate association [KSA] database). The output is the kinase activity inferred from phosphoproteomics data.

## Supplementary Information

Additional file 1: Figures S1–S7. Supplementary figures supporting the bimodality model development, benchmarking, pan-cancer TABP analyses, TROP2 bimodality validation, and immune-signaling characterization. Fig. S1. Effect of changing the relative weights and mean separation for a mixture of Gaussians used for the bimodality model. Fig. S2. Benchmarking data set of 100 known bimodal and unimodal distributions used to evaluate BI and BIC diff performance. Fig. S3. Performance of BI and BIC diff in identifying bimodal distributions. Fig. S4. KEGG pathway enrichment of indication-specific TABPs and Venn analysis of pan-cancer TABPs. Fig. S5. TROP2 bimodality in the CPTAC COAD cohort, including external validation in the T1NxM0 CRC and TCGA CRC cohorts. Fig. S6. TROP2 bimodality in COAD with demographic and clinical features. Fig. S7. Pathway and immune-signaling analyses linked to TROP2 bimodality

Additional file 2: Tables S1–S19. Bimodality benchmark and CPTAC pan-cancer bimodality scores. Each table corresponds to one sheet of the spreadsheet, as listed below. Table S1. Benchmarking of the bimodality index across sample sizes. Table S2. Benchmarking of the BIC difference across sample sizes. Table S3. Tumor-associated bimodal proteins across cohorts. Table S4. CPTAC BRCA tumor cohort bimodality scores. Table S5. CPTAC ccRCC normal cohort bimodality scores. Table S6. CPTAC ccRCC tumor cohort bimodality scores. Table S7. CPTAC COAD normal cohort bimodality scores. Table S8. CPTAC COAD tumor cohort bimodality scores. Table S9. CPTAC GBM tumor cohort bimodality scores. Table S10. CPTAC HNSCC normal cohort bimodality scores. Table S11. CPTAC HNSCC tumor cohort bimodality scores. Table S12. CPTAC LSCC normal cohort bimodality scores. Table S13. CPTAC LSCC tumor cohort bimodality scores. Table S14. CPTAC LUAD normal cohort bimodality scores. Table S15. CPTAC LUAD tumor cohort bimodality scores. Table S16. CPTAC OV tumor cohort bimodality scores. Table S17. CPTAC PDAC normal cohort bimodality scores. Table S18. CPTAC PDAC tumor cohort bimodality scores. Table S19. CPTAC UCEC tumor cohort bimodality scores

Additional file 3: Tables S20–S27. Pathway enrichment, pan-cancer TABPs, and TROP2 differential analyses. Each table corresponds to one sheet of the spreadsheet, as listed below. Table S20. Pathway enrichment analysis of the 2401 TABPs. Table S21. Pan-cancer TABPs identified in at least three indications. Table S22. TROP2-high vs TROP2-low differential protein expression in COAD. Table S23. TROP2-high vs TROP2-low differential RNA-seq expression in COAD. Table S24. MOFA multi-omics factor analysis in COAD. Table S25. Functional annotation of TABPs. Table S26. Reference gene sets used in the analyses. Table S27. Power analysis across mixing weights and separation strengths

## Data Availability

CPTAC processed RNA-seq, proteomics, phosphoproteomics and clinical data, preprocessed according to the common data analysis pipeline [87] that minimizes differences between data generated in different laboratories. Processed omics data and clinical metadata were downloaded from LinkedOmics [76, 77, 88] for LUAD: https://www.linkedomics.org/data_download/CPTAC-LUAD/, LSCC: https://www.linkedomics.org/data_download/CPTAC-LSCC/, OV: https://www.linkedomics.org/data_download/CPTAC-OV/, GBM: https://www.linkedomics.org/data_download/CPTAC-GBM/, PDAC: https://www.linkedomics.org/data_download/CPTAC-PDAC, HNSCC: https://www.linkedomics.org/data_download/CPTAC-HNSCC/, UCEC: https://www.linkedomics.org/data_download/CPTAC-UCEC/, BRCA: https://www.linkedomics.org/data_download/CPTAC-BRCA/, COAD: https://www.linkedomics.org/data_download/CPTAC-COAD/, and ccRCC: https://www.linkedomics.org/data_download/CPTAC-CCRCC/. The clinical data used in the portal were collected from CPTAC with the May 2022 update. Age was truncated to 90 years. Tumors with a size of ≤0 cm were replaced with “NA” (not applicable). CPTAC immune-related information was downloaded from Petralia et al [65]: https://doi.org/10.1016/j.cell.2024.01.027. TCGA processed proteomics data from colon and rectal cancer were downloaded from Zhang et al [43, 89]: https://doi.org/10.1038/nature13438 (Supplementary Information S4). Processed proteomics data from T1NxM0 colorectal cancer were downloaded from Zhuang et al [59, 90]: https://doi.org/10.7554/eLife.82959 (Figure 1—source data 3). Original code has been deposited at Zenodo [91]: https://zenodo.org/records/20447813. Both the deposited data and source code are released under the Creative Commons Attribution 4.0 International (CC-BY-4.0) license.
